# Lithium induces microcysts and polyuria in adolescent rat kidney independent of cyclooxygenase‐2

**DOI:** 10.1002/phy2.202

**Published:** 2014-01-22

**Authors:** Gitte Kjaersgaard, Kirsten Madsen, Niels Marcussen, Boye L. Jensen

**Affiliations:** 1 Department of Cardiovascular and Renal Research, University of Southern Denmark, Odense, Denmark; 2 Department of Pathology, Odense University Hospital, Odense, Denmark

**Keywords:** Aquaporin, prostaglandin, vasopressin

## Abstract

In patients, chronic treatment with lithium leads to renal microcysts and nephrogenic diabetes insipidus (NDI). It was hypothesized that renal cyclooxygenase‐2 (COX‐2) activity promotes microcyst formation and NDI. Kidney microcysts were induced in male adolescent rats by feeding dams with lithium (50 mmol/kg chow) from postnatal days 7–34. Lithium treatment induced somatic growth retardation, renal microcysts and dilatations in cortical collecting duct; it increased cortical cell proliferation and inactive pGSK‐3*β* abundance; it lowered aquaporin‐2 (AQP2) protein abundance and induced polyuria with decreased ability to concentrate the urine; and it increased COX‐2 protein level in thick ascending limb. Concomitant treatment with lithium and a specific COX‐2 inhibitor, parecoxib (5 mg/kg per day, P10–P34), did not prevent lithium‐induced microcysts and polyuria, but improved urine concentrating ability transiently after a 1‐desamino‐8‐D‐arginine vasopressin challenge. COX‐2 inhibition did not reduce cortical lithium‐induced cell proliferation and phosphorylation of glycogen synthase kinase‐3*β* (GSK‐3*β*). COX‐1 protein abundance increased in rat kidney cortex in response to lithium. COX‐1 immunoreactivity was found in microcyst epithelium in rat kidney. A human nephrectomy specimen from a patient treated for 28 years with lithium displayed multiple, COX‐1‐immunopositive, microcysts. In chronic lithium‐treated adolescent rats, COX‐2 is not colocalized with microcystic epithelium, mitotic activity, and inactive pGSK‐3*β* in collecting duct; a blocker of COX‐2 does not prevent cell proliferation, cyst formation, or GSK‐3*β* inactivation. It is concluded that COX‐2 activity is not the primary cause for microcysts and polyuria in a NaCl‐substituted rat model of lithium nephropathy. COX‐1 is a relevant candidate to affect the injured epithelium.

## Abstract (precis)

Long‐term use of lithium is associated with development of microcysts in the kidney. In this study the role for cyclooxygenase‐2 (COX‐2)‐derived prostaglandins in cyst formation was tested in a rat model. Inhibition of COX‐2 did not resolve or prevent kidney injury. COX‐1 was associated with the cyst epithelium and is more likely to play a functional role.

## Introduction

Lithium has been used for decades to treat patients with bipolar affective disorder. Treatment causes a range of renal adverse effects: polyuria, microcysts, and, more infrequently, a decrease in glomerular filtration rate (GFR) and renal fibrosis (Hestbech et al. 1977; Boton et al. 1987; Farres et al. 2003). Data from our laboratory have shown that the postnatal period in rats around weaning constitutes a sensitive “window” compared to adult kidney, where lithium treatment precipitates significant morphological alterations with cortical epithelial proliferation and microcysts that resemble the human counterpart (Kjaersgaard et al. 2012). Glycogen synthase kinase‐3*β* (GSK‐3*β*) is a known target of lithium (Klein and Melton 1996; Stambolic et al. 1996), and lithium‐induced microcysts are GSK‐3*β* positive and the cyst epithelium is proliferative. Lithium treatment in rodents, like in patients, leads to polyuria (Christensen et al. 1982; Rao et al. 2005). The question then arises whether the observed cysts are directly mediated by lithium in target cells or induced secondarily by, for example, paracrine mediators and/or the accompanying polyuria. Prostaglandins are thought to promote cyst growth in polycystic kidney disease and in the rat kidney cyclooxygenase‐2 (COX‐2) is expressed in cells of the thick ascending limb of Henles loop (TAL) and in the macula densa in cortex and in medullary interstitial cells (Harris et al. 1994). The main product of COX‐2 in the kidneys is prostaglandin E_2_ (PGE_2_), which is known to promote salt and water excretion after lithium (Bonvalet et al. 1987; Breyer and Breyer 2000). Casuistic reports show that lithium‐induced nephrogenic diabetes insipidus (NDI) can be attenuated by nonsteroidal antiinflammatory drugs (NSAIDs), which are COX inhibitors, in patients (Lam and Kjellstrand 1997). In animal models of NDI induced by lithium, increased expression of COX‐2 and elevated urinary excretion of PGE_2_ has been shown (Kotnik et al. 2005; Rao et al. 2005). Inhibition of COX‐2 attenuates lithium‐induced polyuria in adult mice and rats (Sugawara et al. 1988; Kim et al. 2008). Mice lacking prostaglandin E_2_ synthase‐1 (mPGES‐1) are resistant to lithium‐induced polyuria (Jia et al. 2009). It is therefore possible that COX‐2/PGE‐S/PGE_2_‐driven polyuria contributes to the development of microcysts. Because increased flow per se is not typically associated with microcysts, a direct effect of lithium on the epithelium which could be amplified by prostaglandins is also possible. Lithium decreases GSK‐3*β* enzyme activity by phosphorylation at the serine 9 residue (Sutherland et al. 1993) and pGSK‐3*β* is associated with microcyst epithelium (Kjaersgaard et al. 2012). GSK‐3*β* inhibition could therefore be implicated in the cystic changes seen after lithium treatment. As COX‐2 is upregulated in cortex in conditions with negative salt balance and polyuria as, for example, induced by furosemide (Mann et al. 2001) or lithium (Rao et al. 2005), the epithelium is likely exposed to elevated concentration of prostaglandins. The PGE_2_‐EP_4_ receptor is localized in the nephron segments injured after postnatal lithium treatment (Jensen et al. 2001; Kjaersgaard et al. 2012). Activation of the EP_4_ receptor can lead to PI3 kinase – PKB/Akt‐mediated phosphorylation and inactivation of GSK‐3*β* (Fujino et al. 2002). Activation of the EP_4_ receptor leads to increased formation of cyclic adenosine monophosphate, which is known to be involved in cyst formation in polycystic kidney disease (Belibi et al. 2004). Therefore, an increased formation of prostaglandins in cortex could promote aberrant cystic epithelial changes after lithium treatment. It was hypothesized that lithium treatment leads to renal microcysts through COX‐2 activity. To address this, lithium was administered to rats in the sensitive postnatal “window” (postnatal days 7–34), with and without the selective COX‐2 inhibitor parecoxib. Kidney tissue, plasma, and salt and water metabolism were analyzed. In addition, aquaporin‐2 (AQP2) protein was followed as a differentiation marker for principal cells.

## Materials and Methods

### Animals

All animal experiments conformed to the Danish national guidelines for the care and handling of animals and to the published guidelines from the National Institutes of Health. Experiments were approved by the national Danish Animal Experiments review board under The Department of Justice (approvals no. 561‐1050 [period 2005–2010] and 561‐1875 [period 2010–2015]) and by the Review Board at the Faculty of Health Science, Animal Facility, University of Southern Denmark. Animals were housed at the Biomedical Laboratory at University of Southern Denmark. Animals were kept at a 12:12 h light:dark cycle and provided with free access to standard pathogen‐free rat chow (Altromin cat.log 1310, Lage, Germany; Na^+^ 2 g/kg, Cl^−^ 5 g/kg) and tap water. Female Wistar rats were used for breeding.

#### Series 1

Kidney tissue was stored from a published series of rats (Kjaersgaard et al. 2012) with lithium treatment from P7 to P28 (50 mmol/kg diet).

#### Series 2

Litters were reduced to eight pups (males only were investigated) to ensure equal feeding. Half of the dams were kept on standard rat chow, whereas the other half was provided with lithium‐enriched chow (50 mmol/kg diet) from postnatal day 7 (P7). Animals had free access to tap water, isotonic saline, and a solid salt lick (100% NaCl). One half of each litter was injected subcutaneously (SC) with Parecoxib (2.5 *μ*g/g in 5 *μ*L/g isotonic glucose twice daily) and the other with vehicle (5 *μ*L/g isotonic glucose twice daily) from P10. Thus, animals were divided into four groups: control‐vehicle (CV), control‐parecoxib (CP), lithium‐vehicle (LV), and lithium‐parecoxib (LP). A total of 10 pups were utilized in each group. At postnatal day 29, the adolescent rats were placed in metabolic cages (*N* = 2 from each of the four groups per experiment, repeated five times). Animals had free access to water and lithium‐enriched or standard granulate chow. To avoid food contamination in the urine and feces collections, diet in the metabolic cages was administered as 1.7 mm granules. Granules were produced from pulverized pellets dissolved in 70% ethanol, squeezed through a sieve and dried for a minimum of 3 h (60°C). After a run‐in period of two consecutive days, 24 h urine was collected for baseline measurements before urine concentrating ability was tested with a bolus injection of 1‐desamino‐8‐D‐arginine vasopressin (dDAVP) (1 *μ*g/kg SC). Urine was collected 4 and 6 h post‐dDAVP treatment. On the following day animals were anesthetized by intraperitoneal injections of sodium pentobarbital 50 mg/kg. All rats donated at least one kidney to RNA/protein measurements. Half of the rats were exsanguinated by cardiac puncture to obtain plasma in which case tissue was used from both kidneys for RNA/protein. The other half of the rats donated one kidney for protein/RNA after ligation of the renal artery. The other kidney was then perfusion fixed. Plasma was isolated by centrifugation. Kidneys were separated into cortex and medulla and snap frozen in liquid nitrogen. Fixation was through the left cardiac ventricle with 4% phosphate‐buffered formaldehyde.

### Human kidney tissue

The nephrectomy specimen was obtained as previously described (Kjaersgaard et al. 2012) from a 71‐year‐old male patient treated with lithium for 28 years and undergoing nephrectomy due to renal cell cancer. The patient gave informed written consent to participate, and experiments were approved by the Regional Biomedical Research Ethics Committee (S‐VF‐20010035).

### Osmolality and electrolyte measurements

Plasma and urine osmolalities were determined by freeze‐point depression (Osmomat 030‐D, Gonotec, Bie and Berntsen and The Advanced Osmometer 3D3, Advanced Instruments). Na^+^, K^+^, and Li^+^ concentrations were determined by flame photometry (model IL 943; Instrumentation Laboratory, Lexington, MA).

### Hormone measurements

Urinary PGE_2_ was measured using enzyme immunoassay kit (Cayman, #514010). In brief, urine was used unacetylated, diluted according to pilot experiments (1:5–1:500), and each dilution was measured in duplicate. The amount of PGE_2_ in each sample is inversely proportional to the measured absorbance as the kit is competitive. To each well, antibody specific to PGE_2_ and PGE_2_‐acetylcholinesterase (AChE) was added. These two reagents bind competitively to the anti‐mouse IgG used to coat the well. AChE reacted with Ellman's Reagent and this reaction, giving a yellow color, was then measured spectrophotometrically.

Plasma renin was measured by radioimmunoassay. In brief, plasma samples, standards, and controls were incubated with substrate (enriched sheep angiotensinogen) and primary antibody at 37°C for 24 h. The reaction was then quenched with cold KBC buffer (0.5 mol/L Tris buffer, pH 7.4 with 10 mmol/L titriplex III, 0.25 mmol/L thiomersal, and 1 g/L albumin) before tracer (^125^I‐angiotensin I, diluted 1:7 in buffer) was added and samples were incubated overnight at 4°C. Finally, samples were suspended in SAC‐CEL (solid‐phase secondary antibody coated cellulose suspension, IDS, U.K.) and separated using centrifugation. The reactivity of the sediment was determined by gamma counting.

### Quantitative polymerase chain reaction

Isolation of RNA from control and lithium‐treated kidney cortex and medulla was carried out using RNeasy mini kit (Qiagen Nordic, Copenhagen, Denmark) according to manufacturer's protocol. RNA was quantified using NanoPhotometer from Implen (AH Diagnostics, Aarhus V, Denmark) and stored at −80°C. cDNA was obtained by reverse transcription of 1 *μ*g total RNA using the iScript cDNA synthesis kit (Bio‐Rad Laboratories, Copenhagen, Denmark). For qPCR, duplicates of cDNA corresponding to 50 ng of total RNA were used as template and mixed with 10 pmol of each primer (COX‐2: 5′‐cgg‐gat‐ccg‐aaa‐tgg‐ctg‐cag‐agt‐tg‐3′; 5′‐atg‐gtg‐gct‐gtc‐ttg‐gta‐3′; 331 bp; COX‐1: 5′‐cgg‐gat‐ccg‐ctg‐ctg‐aga‐agg‐gag‐tt‐3′; 5′‐gga‐att‐cgg‐tgg‐tac‐tgt‐cgt‐tcc‐a‐3′; 188 bp; Glyceraldehyde 3‐phosphate dehydrogenase (nested): 5′‐ctc‐atg‐acc‐aca‐gtc‐cat‐gc‐3′; 5′‐ttc‐agc‐tct‐ggg‐atg‐acc‐tt‐3′; 155 bp), iQ SYBR Green Supermix (Bio‐Rad), and RNase‐free water to a total volume of 25 *μ*L. Water and samples without reverse transcriptase were used as negative controls. After denaturing the mixture for 3 min at 95°C, 40 cycles were run as follows: 30 sec of denaturing at 95°C and 45 sec of annealing and extension at 60°C (MyIQ ICycler; Bio‐Rad). By measuring SYBR green fluorescence in each cycle, amplification was detected. Purified PCR product was used to generate a standard curve by plotting threshold cycle against serial dilution of PCR product of the gene of interest.

### Western blotting

Proteins were homogenized in sucrose/imidazole buffer ([0.3 mol/L sucrose, 25 mmol/L imidazole, 1 mmol/L ethylenediaminetetraacetic acid, pH 7.2]; right before use, the buffer was supplemented with protease inhibitors [0.4 mol/L pefablock and 2.1 mmol/L leupeptin] and phosphatase inhibitors [1 mmol/L Na orthovanadate, 0.2 mol/L NaF, and 0.082 *μ*g/*μ*L okadaic acid]) and quantified by the Bradford method. For western blotting, 10 *μ*g of protein was run on a sodiumdodecyl sulfate polyacrylamide gel electrophoresis (SDS‐PAGE) gel. Each sample was supplemented with reducing agent (NuPAGE Sample Reducing agent; Invitrogen, Taastrup, Denmark) and sample buffer (NuPAGE LDS Sample buffer; Invitrogen) and then denatured for 5 min at 95°C before running. Proteins were transferred to an activated 0.45‐*μ*m pore‐size Immobilon‐P polyvinylidene difluoride membrane (Millipore, Hellerup, Denmark) in the blotting system (XCell SureLock Mini‐Cell system; Invitrogen). Membranes were blocked with 5% nonfat milk in tris buffered saline and tween 20 for 1 h before incubation with primary antibody. Primary antibodies against GSK‐3*β* (9315, Cell Signaling [BioNordika Denmark A/S, Glostrup, Denmark], 1:2500), pGSK‐3*β*‐s9 (9336, Cell Signaling, 1:1000), COX‐2 (sc‐1747, Santa Cruz [Aarhus, Denmark], 1:1000), COX‐1 (160109, Cayman [Aarhus, Denmark], 1:1000), AQP2 (sc‐9882, Santa Cruz, 1:2000), and *β*‐actin (ab8227, Abcam [Cambridge, UK], 1:20000) were used. Secondary antibodies used were P0448 and P0449 (both 1:2000, Dako, Glostrup, Denmark). The labeling was visualized by the enhanced chemiluminiscence plus Western Blotting Detection System (Amersham Biosciences/GE Healthcare Europe GmbH, Brondby, Denmark).

### Immunohistochemistry

Tissue sections were deparaffinized in Tissue Clear and rehydrated in a graded series of ethanol (99–70%). Antigen retrieval was carried out by boiling for 20 min in 1× Target Retrieval Solution Citrate buffer (Dako). Primary antibodies against COX‐2 (sc‐1747, Santa Cruz, 1:500), COX‐1 (160109 Cayman, 1:500), and AQP2 (sc‐9882, Santa Cruz, 1:50) were used. The antigen–antibody complex was visualized by horseradish peroxidase‐conjugated secondary antibody (EnVision ready‐to‐use polymer, P0449 1:1000 or 1:200, Dako). After washing, the labeling was visualized with 3,3′‐diaminobenzidine (DAB+, K3468, Dako). The reaction was quenched with water and the slides were counterstained with Mayer's Hematoxylin for 2 min. For visualization of tissue damage, deparaffinized sections were stained with hematoxylin and eosin, rehydrated, and mounted.

### Statistics

All values are presented as means ± standard error of means (SEM). Two groups were compared by unpaired Student's *t*‐test. Multiple comparisons were analyzed with two‐way analysis of variance (ANOVA) followed by Bonferroni's multiple comparison test or with the nonparametric Kruskal–Wallis followed by Dunn's multiple comparisons test. When necessary, data were log transformed to allow for parametric testing. *P* < 0.05 (two‐sided value, *) was considered significant. *P* < 0.01 was designated **. All statistical tests were performed using Graph Pad Prism 5.01.

## Results

### Effect of lithium and parecoxib treatment on kidney tissue COX‐2 abundance

**Figure 1.**
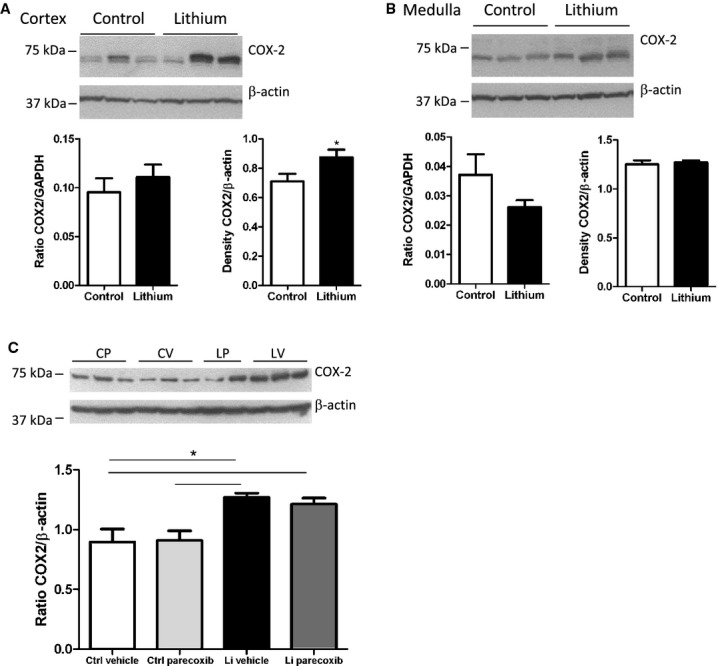
Effect of lithium treatment on COX‐2 mRNA and protein level. (A) Effect of lithium treatment through postnatal days 7–28 on kidney cortex COX‐2 mRNA level (left panel) and cortex COX‐2 protein abundance (right panel and immunoblottings shown above). Values are mean ± SEM, * *P* < 0.05 (*n* = 6 for control, *n* = 12 for lithium treatment). (B) Effect of lithium treatment P7–P28 on kidney medulla COX‐2 mRNA level (left panel) and COX‐2 protein abundance (right panel, immunoblottings shown above). Values are mean ± SEM, * *P* < 0.05 (*n* = 6 for control, *n* = 12 for lithium treatment). (C) Protein abundance of COX‐2 in kidney cortex as assessed by western blotting of tissue from rats treated with vehicle (control‐vehicle, CV); parecoxib, 5 mg/kg per day (control‐parecoxib, CP); lithium and vehicle (Li‐vehicle, LV); and lithium and parecoxib, 5 mg/kg per day (Li‐parecoxib, LP). Data are presented as means ± SEM, *P < 0.05; *n* = 5 in all groups.

Kidney tissue from rats treated with lithium from P7–28 (series 1) showed no significant difference in the level of COX‐2 mRNA, whereas COX‐2 protein abundance was increased significantly in cortex (Fig. 1A). COX‐2 was not changed in medulla (Fig. 1B). Lithium treatment in series 2 (P7–P34) led to a significant increase in COX‐2 protein level compared to control (Fig. 1C). Parecoxib treatment did not alter COX‐2 protein abundance compared to vehicle in lithium‐treated rats and in control rats (Fig. 1C). The abundance of *β*‐actin protein did not change significantly with any condition and was used accordingly to normalize for loading and quality.

### Effect of COX‐2 inhibition on lithium‐induced kidney tissue

**Figure 2.**
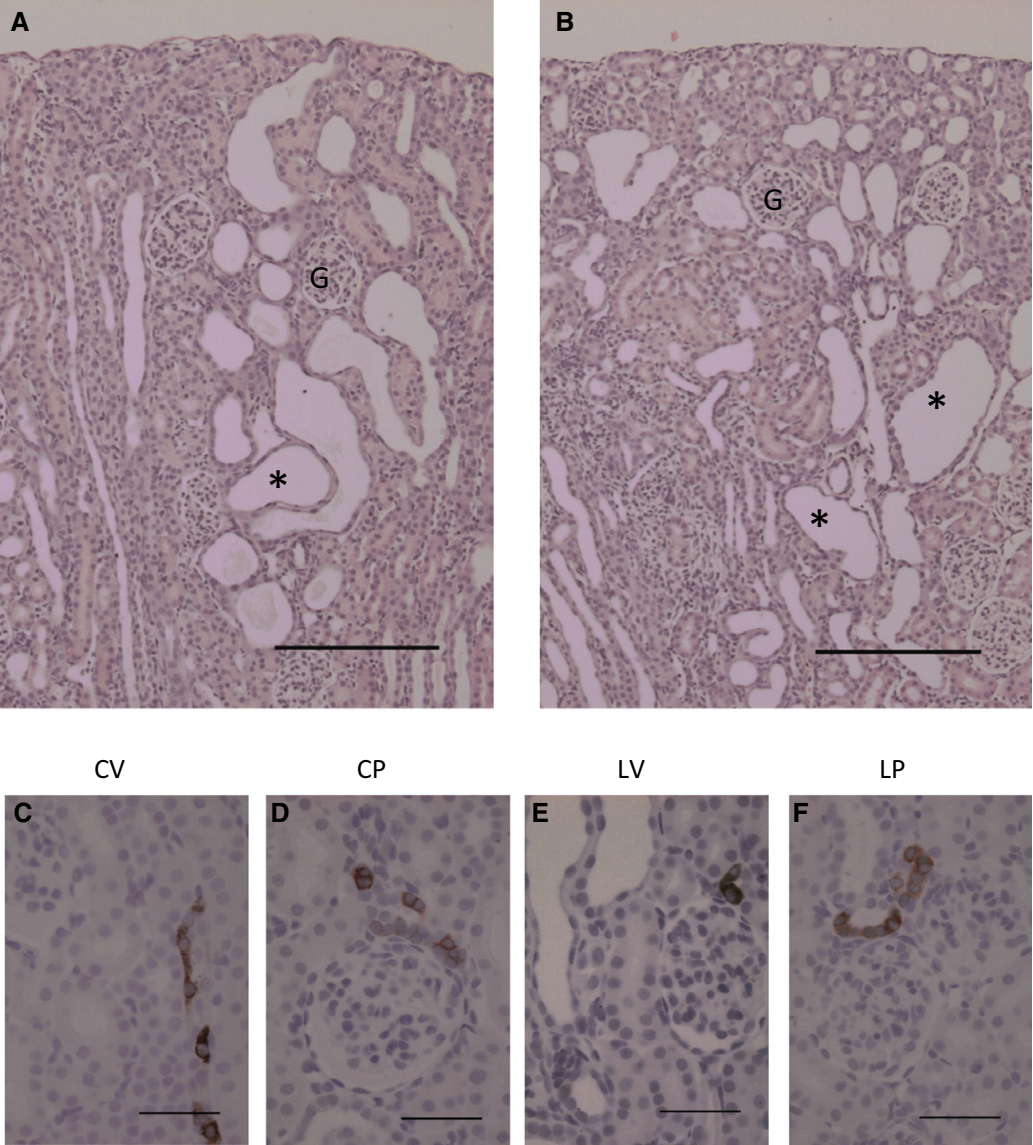
Effect of lithium with and without a COX‐2 inhibitor on kidney morphology and COX‐2 localization. (A and B) Hematoxylin and eosin stained kidney sections from rats treated with lithium (P7–P34) without (A) and with parecoxib (B, 5 mg/kg per day, P10–P34). Parecoxib treatment itself had no visible effect on kidney morphology compared to vehicle (not shown). Lithium‐treated kidneys displayed tubular dilatations and microcysts (*) in medullary rays (A) which were not altered visibly by parecoxib (B). “G” ‐ glomerulus four different kidneys were inspected for each group. Scale bar = 200 *μ*m. (C–F), COX‐2 labeling was located primarily to the macula densa region in all four groups. The dilated tubules and microcysts in no case were COX‐2 positive (E and F). Scale bar = 50 *μ*m.

Lithium treatment P7–34 led to structural kidney tissue changes (Fig. 2A and B). The kidney cortex displayed dilated and microcystic tubules associated with medullary rays, whereas the outer and inner medulla did not exhibit dilatations or cysts. The microcysts appeared to originate from collecting ducts (“*” in Fig. 2A and B). By simple inspection, treatment with the selective COX‐2 inhibitor parecoxib did not reverse or attenuate the lithium‐induced microcystic tissue alterations (Fig. 2A – vehicle and B – parecoxib). Parecoxib alone had no detectable effect on kidney morphology (not shown). Immunohistochemical labeling of control kidneys for COX‐2 showed distinct signals associated with the TAL and in particular the macula densa (Fig. 2C and D). Parecoxib treatment did not alter the distribution of COX‐2 (Fig. 2C and D) compared to control. Lithium treatment with or without parecoxib did not alter the association of COX‐2 with the cortical loop of Henle (Fig. 2E and F) compared to control, and there were no major changes in staining intensity (Fig. 2C and D vs. E and F). Dilated tubules and regular microcysts in cortical medullary rays were not immunopositive for COX‐2 (Fig. 2E and F).

### Effect of COX‐2 inhibition on somatic growth and plasma parameters

Rats treated with lithium P7–34 displayed reduced somatic and kidney growth, but relative kidney hypertrophy with increased kidney weight/body weight ratio compared to control rats (Table 1). Parecoxib treatment had no effect on growth in either the control or the lithium group. Lithium reached concentrations of ~1 mmol/L in plasma and there was no difference in plasma lithium concentration between LV and LP groups (Table 1). Lithium was not detectable in plasma from controls (Table 1). Plasma osmolality tended to be higher in lithium‐treated rats, however, this only reached a significant level in the parecoxib–lithium group (Table 1). Plasma sodium concentration was higher in lithium‐treated animals compared to the other three groups (Table 1). Hematocrit and plasma K^+^ concentration were not significantly different between groups (Table 1). Lithium treatment increased plasma renin concentration significantly (Table 1) and this increase was blocked by parecoxib (Table 1). Cortical tissue PGE_2_ concentrations were not different between the four groups (Table 1).

**Table 1.** Weight and plasma data at euthanasia.

	CV	CP	LV	LP
Body weight (g)	118.3 ± 2.4	111.8 ± 2	52.2 ± 2.7*	49.7 ± 2.2*
Kidney weight (mg)	1121.0 ± 20.4	1186.8 ± 50.4	764.0 ± 37.8*	745.25 ± 3.7*
Kidney weight/body weight (mg/g)	9.9 ± 0.1	10.6 ± 0.3	15 ± 0.9*	14.5 ± 1.6*
Plasma osmolality (mOsm/kg H_2_O)	294 ± 0.6	294 ± 2.2	310 ± 3.4*	313 ± 3.6*
Plasma [Na^+^] (mmol/L)	137.2 ± 1.0	137.9 ± 0.7	146.4 ± 2.0*	145.0 ± 2.7
Plasma [K^+^] (mmol/L)	5.6 ± 0.3	5.5 ± 0.3	5.3 ± 0.2	5.5 ± 0.6
Plasma [Li^+^] (mmol/L)	ND	ND	0.95 ± 0.14	1.29 ± 0.13
Hematocrit (%)	34.8 ± 0.8	35.2 ± 0.8	32.2 ± 1.0	31.8 ± 1.2
Plasma renin ng (ANG I/L/h)	2442 ± 388	3242 ± 385	7483 ± 894*	6799 ± 3343
Cortical PGE_2_ (pg/mg protein)	22.5 ± 1.5	19.2 ± 1.3	32.1 ± 5.3	23.7 ± 4.8

Because plasma was not sampled for rats used for fixation, *n* = 9–10 for body weight and cortical PGE_2_ data, while all plasma data are *n* = 4–5. Data have been analyzed with one‐way ANOVA followed by Bonferroni's multiple comparisons test. Data are presented as means ± SEM. Small groups (*n* < 5) were analyzed with nonparametric Kruskal–Wallis test followed by Dunn's multiple comparisons test. ND, not determined.

* *P* < 0.05 compared to the CV and CP groups.

* *P* < 0.05 CV and CP versus LV.

### Effect of COX‐2 inhibition on lithium‐induced cell proliferation and GSK‐3*β* phosphorylation

**Figure 3.**
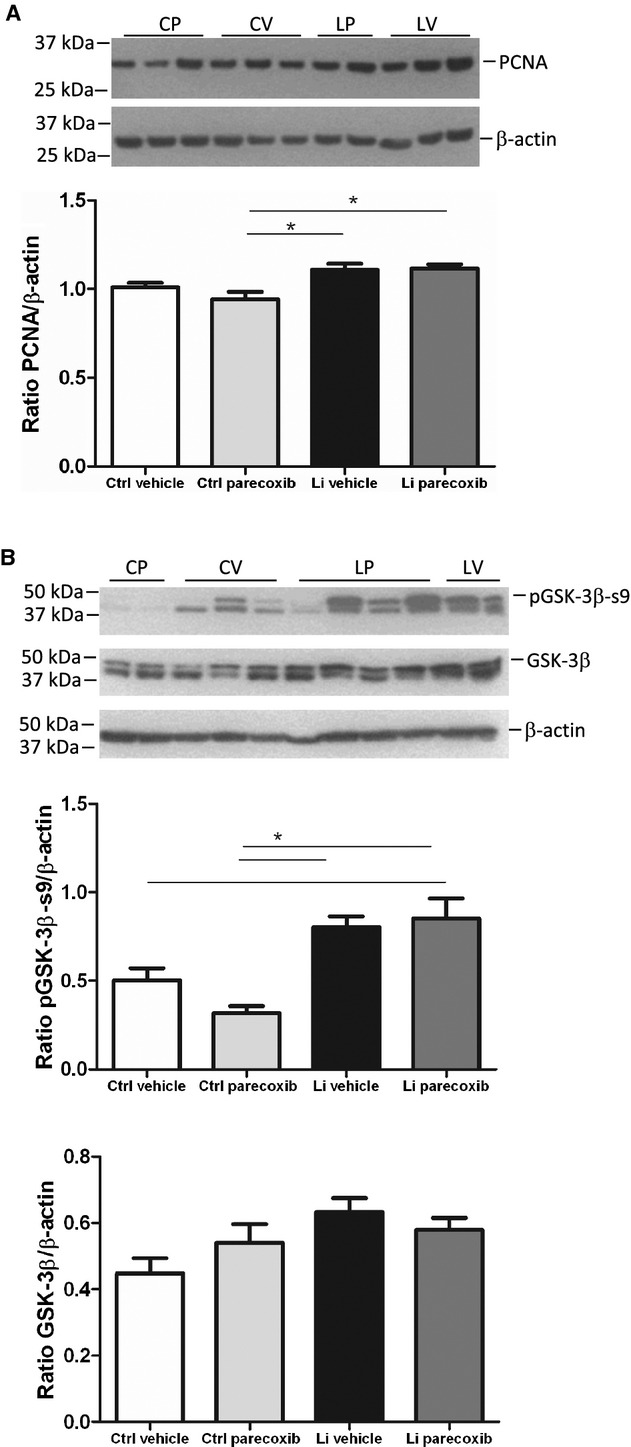
Effect of lithium treatment with and without a COX‐2 inhibitor on cell proliferation and glycogen synthase kinase‐3*β* phosphorylation. (A) Western blotting (upper panel) of kidney cortex tissue homogenates for proliferating cell nuclear antigen (PCNA), a marker of mitosis, and densitometric evaluation of gels (diagram). Values were normalized to *β*‐actin protein abundance. Lithium increased PCNA protein abundance with no effect of parecoxib treatment. Data were analyzed with two‐way ANOVA followed by Bonferroni's multiple comparisons test. The columns show mean values ±SEM, *P < 0.05; *n* = 5 in all groups. (B) Western blotting of kidney cortex tissue homogenates for phospho‐serine 9 (pGSK‐3*β*‐s9) and total GSK‐3*β* (upper panel) and densitometric evaluation of gels (diagrams). For samples showing double bands, the upper band was chosen for quantification according to the predicted size of the protein. Lithium treatment significantly increased the abundance of pGSK‐3*β*‐s9. No effect of parecoxib treatment was seen. The abundance of total GSK‐3*β* was not significantly altered between any of the four experimental groups. Data were analyzed with two‐way ANOVA followed by Bonferroni's multiple comparisons test. Data are presented as means ± SEM, *P < 0.05; *n* = 5 in all each group.

Lithium treatment led to a significant increase in protein abundance of the mitosis marker proliferating cell nuclear antigen (PCNA) in cortex compared to the CP group but not vehicle‐control group (Fig. 3A). PCNA abundance in medulla was not different between the groups (not shown). Parecoxib treatment did not alter cortical PCNA abundance in control or lithium‐treated group (Fig. 3A). Lithium treatment led to a significant increase in the abundance of serine 9 phosphorylated inactive GSK‐3*β* protein in kidney cortex compared to CP group but not the CV group (Fig. 3B). Parecoxib treatment did not significantly alter the level of pGSK‐3*β*‐s9 compared to control and to lithium (Fig. 3B). Abundance of total GSK‐3*β* protein was not significantly altered by lithium treatment, by parecoxib treatment, or by combination of the two (Fig. 3B).

### Effect of COX‐2 inhibition on urine concentration ability after lithium treatment

**Figure 4.**
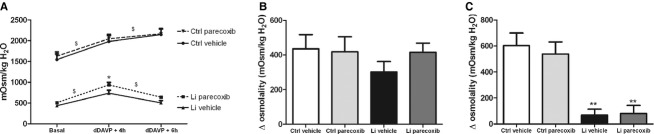
Effect of a COX‐2 inhibitor on lithium‐induced polyuria. (A) At baseline, urine osmolality was significantly higher in control animals compared to lithium‐treated animals. Upon stimulation with dDAVP, all animals increased urinary osmolality, but lithium‐treated animals were not able to sustain the increase at 6 h after dDAVP injection. Lithium‐parecoxib–treated animals had a significantly higher urine osmolality 4 h after dDAVP injection as compared with lithium‐vehicle–treated animals indicated by *. Control and lithium‐treated groups were analyzed separately by two‐way ANOVA to avoid interaction followed by Bonferroni's multiple comparisons test. The effect of dDAVP was significant in both groups, symbolized by ^$^. *Significant difference between lithium‐vehicle and lithium‐parecoxib groups at dDAVP + 4 h, also analyzed by two‐way ANOVA. (B) The increase in urinary osmolality (Δ osmolality) was similar in the four groups 4 h after dDAVP injection compared to the basal level. Data are analyzed with two‐way ANOVA followed by Bonferroni's multiple comparisons test. (C) The increase in urinary osmolality was abolished 6 h after dDAVP injection in lithium‐treated animals. Control animals maintained an increased urinary osmolality compared to baseline and significantly larger increase (Δ osmolality) than in lithium‐treated animals. Data are analyzed with two‐way ANOVA followed by Bonferroni's multiple comparisons test. **P < 0.01 Li groups compared to control‐vehicle and control‐parecoxib. Data are presented as means ± SEM;* n* = 10 in all groups.

Diet intake and feces excretion were significantly lower in lithium‐treated animals, whereas sodium excretion in urine was higher in lithium‐treated rats with no effect of parecoxib (Table 2). Urine flow and water intake were significantly and markedly increased by lithium treatment and was associated with a lower basal urine osmolality; none of these baseline parameters was affected by parecoxib (Table 2 and Fig. 4A). At baseline, urinary PGE_2_ excretion was not significantly different between the four groups (Table 2). Control animals increased significantly urine osmolality compared to baseline 4 and 6 h after a dDAVP bolus injection with no additional effect of parecoxib administration (Fig. 4A). Urine flow was decreased by dDAVP compared to baseline (Table 2). Rats treated with lithium displayed lower urine osmolality at all time points compared to control (Fig. 4A). Lithium‐treated rats increased urine osmolality 4 h after dDAVP injection compared to baseline, and the Δ osmolality increase was similar between groups (Fig. 4B). Absolute urine osmolality value was significantly higher after parecoxib treatment after 4 h (Fig. 4A). Sodium excretion was significantly lowered by parecoxib 4 h after dDAVP in lithium‐treated rats (Table 2). After 6 h, urine osmolality had returned to the low baseline level in lithium groups with no effect of parecoxib (Fig. 4A) and thus Δ osmolality was significantly lower compared to control and control‐parecoxib rats (Fig. 4C). Sodium excretion was similar in all four groups 6 h after dDAVP.

**Table 2.** Urine parameters in rats treated with lithium and/or parecoxib before, during, and after dDAVP injection.

Baseline	CV	CP	LV	LP
Diet intake (g/100 g per 24 h)	16.5 ± 0.2	16.1 ± 0.3	11.6 ± 0.8*	12.2 ± 1.0*
Feces excretion (g/100 g per 24 h)	9.7	10.9	6.5*	4.7*
Water intake (mL/100 g per 24 h)	25.2	24.9	62.9*	54.2*
Urine flow (mL/100 g per 24 h)	10	9	42.8*	35.4*
Urinary sodium excretion (mmol/100 g per 24 h)	0.76 ± 0.04	0.76 ± 0.04	1.08 ± 0.05*	1.02 ± 0.05*
Urine PGE_2_ (ng/100 g per 24 h)	31.9 ± 4.2	79.7 ± 29.2	26.7 ± 3.0	25.2 ± 2.5
dDAVP + 4 h				
Urine flow (mL/100 g per 24 h)	4.3	4.7	19.1*	9
Urinary sodium excretion (mmol/100 g per 24 h)	0.62 ± 0.06	0.54 ± 0.04	1.01 ± 0.21	0.28 ± 0.05*
dDAVP + 6 h				
Urine flow (mL/100 g per 24 h)	5.6	5.7	31.4*	38.8*
Urinary sodium excretion (mmol/100 g per 24 h)	1.33 ± 0.10	1.31 ± 0.09	0.94 ± 0.11	1.22 ± 0.18

Data have been analyzed with two‐way ANOVA followed by Bonferroni's multiple comparisons test. Data are presented as means ± SEM. When no standard error is shown data have been log transformed and the geometric mean is shown. Data that did not achieve normal distribution were analyzed with the nonparametric Kruskal–Wallis test followed by Dunn's multiple comparisons test.

* *P* < 0.05 compared to the CV and CP groups.

* *P* < 0.05 LP versus LV. *N* = 10 unless otherwise stated.

In lithium‐treated rats, urine flow 4 h after dDAVP was markedly lower compared to lithium‐treated rats at baseline (Table 2) and only dDAVP‐treated, LV rats had significantly higher diuresis compared to control rats (Table 2). Thus, parecoxib significantly lowered urine flow in dDAVP‐challenged, lithium‐treated compared to LV rats (Table 2).

In lithium‐treated animals, the urine flow had returned to high baseline levels 6 h after dDAVP injection, whereas control animals still had a lower urine flow at this time point (Table 2).

### Effect of lithium and COX‐2 inhibition on AQP2

**Figure 5.**
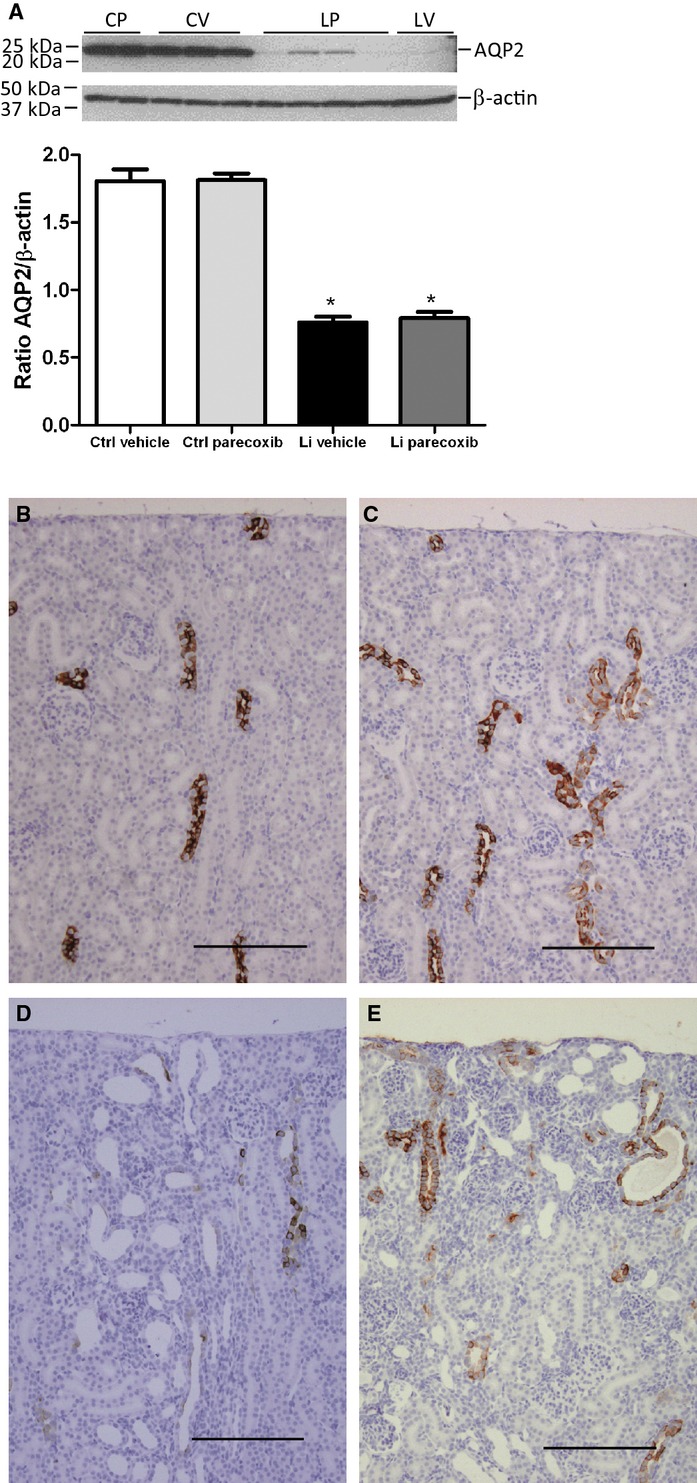
Effect of lithium treatment with and without a COX‐2 inhibitor on AQP2 abundance and localization. (A) The panels show result of western blotting of kidney cortex tissue homogenates for aquaporin‐2 (AQP2, upper panel) and densitometric evaluation of gels (diagram). Values were normalized to *β*‐actin protein abundance. Lithium significantly suppressed the protein abundance of AQP2 with no effect of parecoxib treatment. Columns display mean ± SEM, *P < 0.05, *n* = 6. (B–E) Immunohistochemical labeling of rat kidney sections for AQP2 showed decreased labeling in both lithium‐treated groups (D and E) compared to vehicle and parecoxib‐treated groups (B, C). Parecoxib had no discernible effect on AQP2 signal distribution or intensity (C and E vs. B and D). Dilated tubules and microcysts in lithium‐treated kidneys showed single AQP2 immunopositive cells and cell clusters, but the majority of cells associated with dilatations and cysts were not positive for AQP2 (D and E). Scale bar = 50 *μ*m. Kidney sections from four animals in each group were analyzed. Scale bar = 200 *μ*m.

Next, it was addressed whether AQP2 protein was affected by lithium. Western immunoblotting for AQP2 in vehicle and lithium‐treated rat kidney cortex homogenates displayed a significantly reduced level of both glycosylated AQP2 (not shown) and nonglycosylated AQP2 protein (Fig. 5A) in response to lithium treatment. There was no effect of parecoxib on the lithium‐induced suppression of AQP2 protein level (Fig. 5A). Immunohistochemical labeling of kidney sections for AQP2 showed more widespread localization and more intense immunoreactive protein in control animals compared to lithium‐treated animals (Fig. 5B and D). In lithium‐treated animals, there was a markedly lower number of AQP2‐positive cells in the cortical collecting ducts and most cells associated with regular cysts were AQP2 negative (Fig. 5D and E).

### Effect of lithium treatment on COX‐1 abundance in rat and human kidney

**Figure 6.**
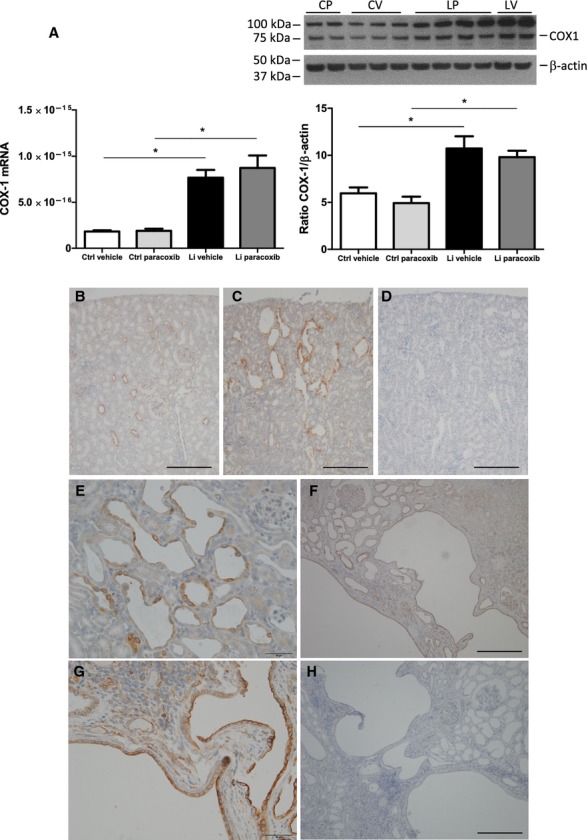
Effect of lithium treatment and COX‐2 inhibition on COX‐1 abundance and localization in rat and human kidney. (A) COX‐1 mRNA expression (left panel) and protein abundance (right panel) in kidney cortex tissue homogenates. Western immunoblotting for COX‐1 with rat kidney cortex homogenate is shown above the diagram. COX‐1 has a molecular weight ~72 kDa and a product at 100 kDa was regularly observed and considered as unspecific. COX‐1 mRNA level and protein abundance were significantly upregulated by lithium with no effect of parecoxib treatment. Columns display mean ± SEM, *P < 0.05 between the groups marked by lines (*n* = 6). (B–E) Immunhistochemical staining of kidney section from control rat (B) and rat treated with lithium (C) for cyclooxygenase‐1 (COX‐1). Cortex is shown. Negative control with omission of primary antibody is seen in (D). At larger magnification of lithium‐treated rat kidney section (E), microcystic and dilated epithelium is COX‐1 positive and in control kidney, immunoreactive COX‐1 is associated with collecting ducts in cortex (B) and medulla (not shown). Scale bar = 200 *μ*m in (B–D) and 50 *μ*m in (E). (F–H) Immunohistochemical staining of kidney sections for COX‐1 from a human nephrectomy specimen removed from a patient treated with lithium for 28 years and suffering from renal clear cell cancer. At low magnification, the sections displayed multiple larger and smaller cysts separated by normally appearing tissue and glomeruli (F). Medulla appeared normal (not shown). Immunopositive reaction was associated with cysts and collecting ducts, whereas the major part of the cortical tissue with proximal convoluted tubules was negative (F and G). At higher magnification, both large and small cysts displayed labeling associated with the epithelial cells that lined the cysts (G). In the absence of primary antibody, the tissue exhibited no reaction (H). Scale bar = 200 *μ*m in (F and H) and 50 *μ*m in (G).

Lithium treatment significantly increased renal cortical tissue level of COX‐1 mRNA and protein in rats (Fig. 6A). Parecoxib had no effect on COX‐1 mRNA and protein abundance (Fig. 6A). In control rat kidneys, COX‐1 was associated with subsets of cells in cortical collecting ducts (Fig. 6B), likely principal cells, as COX‐1 was associated with all cells along the inner medullary collecting duct and with medullary interstitial cells (not shown). The microcysts and dilated collecting ducts in renal cortex from lithium‐treated rats were strongly immunopositive for COX‐1 (Fig. 6C and E). In the absence of primary antibody, no labeling was observed (Fig. 6D). Histological sections of a nephrectomy kidney from a patient treated with lithium for 28 years revealed multiple microcysts and larger lacunar areas lined by epithelium (Fig. 6F). Cysts were observed in cortex (Fig. 6F) but not in medulla (not shown). Focal interstitial fibrosis was seen in the kidney, especially in cortical areas with tubular atrophy. Both normally appearing cortical collecting ducts and the cystic epithelium were immunopositive for COX‐1 (Fig. 6F and G). Cyst epithelium was uniformly positive for COX‐1, whereas in the absence of primary antibody, no reaction was observed by secondary antibody (Fig. 6H).

## Discussion

This study shows lithium‐induced somatic growth retardation with relative kidney hypertrophy, epithelial cell proliferation, and cortical microcysts with significantly increased cortical COX‐2 protein associated with loop of Henle and macula densa. After lithium treatment, rats displayed AQP2 downregulation associated with NDI and reduced urine concentrating ability; increased inactive phopho‐GSK3*β* and increased COX‐1 protein abundance in cortical cystic collecting duct epithelium. While continuous COX‐2 inhibitor treatment transiently and marginally improved dDAVP‐induced urine concentrating ability and lowered plasma renin concentration and improved sodium retention in lithium‐treated rats, it did not alter microcyst formation, AQP2 downregulation, inactivating GSK‐3*β* phosphorylation, and COX‐1 upregulation. Thus, enhanced COX‐2 activity is not likely to be the primary cause for microcysts or diabetes insipidus in the chronic, adolescent rat model of lithium nephropathy. The spatial colocalization with cyst epithelium and upregulation of COX‐1 mRNA and protein abundance in both rat and human kidneys suggest that focus should be on this isoform in relation to potential prostaglandin‐mediated effects on tubular dilatation/microcysts.

The modest increase in COX‐2 protein level and restricted distribution of COX‐2 in TAL/MD segment are similar to previous data from adult rat kidney (Kotnik et al. 2005). The stimulation of COX‐2 by lithium is likely indirect, as no data support that apical lithium uptake occurs in TAL. On the other hand, a low transepithelial transport rate of NaCl in TAL/MD cells, caused by, for example, low dietary NaCl intake or diuretics is a primary stimulus for COX‐2 (Harris et al. 1994; Jensen and Kurtz 1997; Yang et al. 1998). The elevated plasma Na^+^ concentration shows loss of water in excess of sodium. With the moderate stimulation of renin release, extracellular volume of the lithium rats is not severely depleted. Salt supplementation in the present model is necessary to maintain survival, and the support of ECV through the salt lick is the likely cause for the very modest stimulation of renin, cortical COX‐2, and PGE_2_ urine excretion (Christensen and Ottosen 1983; Kjaersgaard et al. 2012). This is also a likely explanation as to why parecoxib had no effect on urine PGE_2_ excretion. The polyuria may have contributed to the significant growth retardation, but rats ate less of the lithium‐containing food pellet per gram weight. Growth retardation is a typical feature of lithium‐treated rodent models and is not likely to cause microcysts and polyuria. Growth impairment is fully reversible; when lithium is withdrawn, rats displayed a rapid catch‐up growth and after 4 weeks without lithium on standard rat chow (postnatal day 70), their weight and baseline renal parameters were normal compared to controls (Kjaersgaard et al. 2012). In mice, COX‐2 expression is increased after 2 weeks of lithium treatment (Rao et al. 2005; Yoshioka et al. 2009). Increased COX‐2 expression after lithium treatment has been shown to correlate with an increased urinary PGE_2_ excretion (Kotnik et al. 2005; Rao et al. 2005; Jia et al. 2009) and an effect of COX‐2 inhibitors (Rao et al. 2005; Kim et al. 2008). While effects on diuresis and AQP2 by lithium were similar to the present observations, the fold change of COX‐2 expression in the mouse models was much larger compared to the present rat model which could be due to the lack of salt substitution and larger extracellular volume depletion. In this study, lithium‐mediated downregulation of AQP2 abundance was confirmed (Marples et al. 1995; Kortenoeven et al. 2012) and no rescue was observed using a COX‐2 inhibitor. This supports the observation by Kortenoeven et al. (2012) that the Li^+^‐mediated AQP2 downregulation is independent of COX‐2. Undisputedly, in situations with marked stimulation of renal prostaglandin formation, as measured by the surrogate marker of urine excretion (Rao et al. 2005; Kim et al. 2008), a contribution of COX‐2 to polyuria and AQP2 downregulation is revealed by pharmacological inhibitors (Norregaard et al. 2007). Similar to these observations, in this study, a transiently increased urine osmolality and reduction in urine flow, plasma renin, and sodium excretion by COX‐2 inhibition were observed in the lithium‐treated group. These findings show that parecoxib reached its target as previously seen with this dose (Stubbe et al. 2003; Norregaard et al. 2005). As COX‐1 is the predominant isoform in collecting ducts and COX‐1 protein abundance was significantly increased in the present rat model, it is likely that COX‐1 activity masks the effects of COX‐2 inhibition and thus explains the present observation of similar PGE_2_ urine excretion in all four groups (Campean et al. 2003). In this study, the dose of parecoxib (5 mg/kg per day) was identical to previous studies in both young and adult animals (Stubbe et al. 2003, 2007; Norregaard et al. 2005). Parecoxib is a prodrug of valdecoxib, which is a potent and selective COX‐2 inhibitor with ED_50_ = 0.03 mg/kg (Gierse et al. 2005). The same dose and route of administration has been used with effect in adult Munich‐Wistar rats (Norregaard et al. 2005) and in Sprague–Dawley rats during kidney development (Stubbe et al. 2003). In the rat study by Kim et al. (2008), a COX‐2 blocker improved baseline diuresis, but was used at 40 mg/kg. At this dose, it is likely also to affect COX‐1 and COX abundances in kidney tissue were not reported (Kim et al. 2008). It cannot be ruled out that the beneficial effects in this study could be caused by COX‐1‐mediated effects and/or a decrease in GFR also could contribute.

Disruption of *α*ENaC prevents lithium‐induced polyuria in mice (Christensen et al. 2011). ENaC is colocalized with dilations/microcysts, mitotic activity and increased expression of pGSK‐3*β*‐s9 in collecting duct after lithium treatment (Kjaersgaard et al. 2012). As no effect of the COX‐2 inhibitor on these parameters was discovered and COX‐2 was not colocalized with the lesions, the cystic change is likely caused by apical entry of lithium and not by paracrine action of COX‐2. Polyuria per se is not likely to have caused the lesions, as in adult, lithium‐treated animals, inhibition of polyuria with large doses of vasopressin does not abolish the lithium‐induced kidney injury (Ottosen et al. 1988). Polyuria by other causes, for example, deletion of sodium potassium 2‐chloride co‐transporter or renal outer medullary potassium channel causes a hydronephrosis‐like phenotype with papillary atrophy and cortical thinning that does not mimic the present cortical microcysts induced by lithium (Takahashi et al. 2000; Lorenz et al. 2002). Similar data have been obtained in mice with inducible AQP2 knockdown (“inducible diabetes insipidus”) that exhibit only mildly dilated cortical and medullary collecting ducts, but no microcysts as seen with lithium (Yang et al. 2006). In summary, lithium treatment of NaCl‐supplemented male rats immediately before and after weaning leads to four times higher diuresis, significant dilatations, and microcysts in cortical collecting ducts and marginal stimulation of COX‐2 abundance in macula densa/loop of Henle segment with no change in PGE_2_ excretion. Concomitant treatment with lithium and a COX‐2 inhibitor yielded a transient and minor improvement in dDAVP‐induced urine concentration, whereas it did not attenuate microcyst formation, GSK3*β* inactivation, and cell proliferation. It is concluded that COX‐2 activity is not responsible for cortical collecting duct cell proliferation and microcysts during chronic lithium treatment.

## Acknowledgments

The authors thank Inger Nissen, Lis Teusch, Kristoffer Rosenstand, Susanne Hansen, and Bodil Kristensen for expert technical assistance.

## Conflict of Interest

None declared.
